# Prevalence of latent tuberculosis infection among participants of the national LTBI screening program in South Korea – A problem of low coverage rate with current LTBI strategy

**DOI:** 10.3389/fpubh.2022.1066269

**Published:** 2023-01-19

**Authors:** Hyung Woo Kim, Jinsoo Min, Joon Young Choi, Ah Young Shin, Jun-Pyo Myong, Yunhee Lee, Hyeon Woo Yim, Hyunsuk Jeong, Sanghyuk Bae, Hoyong Choi, Hyekyung In, Ahyoung Park, Miri Jang, Hyeon-Kyoung Koo, Sung-Soon Lee, Jae Seuk Park, Ju Sang Kim

**Affiliations:** ^1^Division of Pulmonary and Critical Care Medicine, Department of Internal Medicine, College of Medicine, Incheon St. Mary's Hospital, The Catholic University of Korea, Seoul, Republic of Korea; ^2^Division of Pulmonary and Critical Care Medicine, Department of Internal Medicine, College of Medicine, Seoul St. Mary's Hospital, The Catholic University of Korea, Seoul, Republic of Korea; ^3^Department of Occupational and Environmental Medicine, College of Medicine, Seoul St. Mary's Hospital, The Catholic University of Korea, Seoul, Republic of Korea; ^4^Department of Preventive Medicine, College of Medicine, The Catholic University of Korea, Seoul, Republic of Korea; ^5^Division of Tuberculosis Prevention and Control, Korea Disease Control and Prevention Agency, Cheongju, Republic of Korea; ^6^Division of Pulmonary and Critical Care Medicine, Department of Internal Medicine, Ilsan Paik Hospital, Inje University College of Medicine, Busan, Republic of Korea; ^7^Division of Pulmonary and Critical Care Medicine, Department of Internal Medicine, Dankook University College of Medicine, Cheonan, Republic of Korea

**Keywords:** latent tuberculosis infection, prevalence, national tuberculosis control, risk factors, tuberculosis prevention

## Abstract

**Background:**

The Government of South Korea launched a national preemptive latent tuberculosis infection (LTBI) screening program in 2016, including more than 1. 6 million population in congregate settings. The objective of this study was to analyze LTBI prevalence and its risk factors in each setting. Additionally, the proportion of LTBI pool covered by the current national LTBI strategy was investigated.

**Methods:**

Database for results of interferon gamma release assay (IGRA), X-ray, and baseline demographic information was linked with National Health Information Database, national tuberculosis (TB) surveillance database, and national contact investigation database. Participants were categorized into three groups: Group A, workers of postpartum care centers, social welfare facilities and educational institutions; Group B, first year students in high school and out-of-school youths; and Group C, inmates of correctional facilities. Relative risks of LTBI by sex, age, place of living, income level, and comorbidities were calculated.

**Results:**

A total of 444,394 participants in Group A, 272,224 participants in Group B, and 11,511 participants in Group C who participated in the national LTBI screening program between 2017 and 2018 were included, with LTBI prevalence of 20.7, 2.0, and 33.2%, respectively. Age was the single most important risk factor in Group A and Group C. Low-income level was another risk factor commonly identified in all groups. Among participants with positive IGRA results, 2.7, 4.4, and 3.3% in Groups A, B and C, respectively, had past TB exposure history since 2013. Current LTBI guideline targeting high or moderate TB risk disease covered 6.5, 0.6, and 1.1% of participants with positive IGRA results in Groups A, B and C, respectively.

**Conclusion:**

Only a small proportion of participants with positive IGRA results could be covered by the current LTBI strategy. Expansion of LTBI strategy by identifying further high-TB risk group in the general population is required.

## 1. Introduction

Tuberculosis (TB) is still a global threat in the COVID-19 pandemic. Although there has been a large drop in TB notification worldwide, TB death has increased due to reduced access to TB services. In 2020, ~1.5 million deaths were attributable to TB worldwide (1). WHO's END TB Strategy targets an 80% reduction in TB incidence by 2030, with a milestone of 20% reduction by 2020. However, only 11% reduction was achieved globally by 2020 (1).

Approximately one-fourth of total population were infected with *Mycobacterium Tuberculosis* globally (2). The role of management of latent tuberculosis infection (LTBI) in addition to active TB has been underscored for TB elimination (3). In United Nation's high-level meeting held in 2018, a target of providing LTBI treatment for more than 30 million population worldwide by 2022 was suggested (1). Following WHO's guideline for LTBI (4), the national LTBI screening program in South Korea has been expanded since 2010s when the TB incidence decreased to below 100 cases per 100,000 population (5). Considering that the prevalence of human immunodeficiency virus (HIV) is relatively low in South Korea (0.02% of the national population) (6), contact investigation have been a pillar of the national LTBI screening program, which has been fully implemented since 2013. Especially, considering the large gap in TB burden by age group (7), protecting young generation from TB exposure has been an important strategy. However, several TB outbreaks in congregate settings such as schools, postpartum care centers, daycare centers or military units have become important social issues. Indeed, in early 2010s, when TB incidence by age was plotted, the first peak was identified among people in their 20's, suggesting that there might be an ongoing transmission among young generation in the community (8).

With these backgrounds, the government of South Korea launched a “TB-free Korea” program in 2016, including preemptive LTBI screening for more than 1.6 million population in congregate settings (9). In this study, we analyzed LTBI prevalence and its risk factors in each setting. Additionally, by linking with databases of TB contact, we investigated the proportion of LTBI pool covered by the current national LTBI strategy targeting TB contacts and patients with high or moderate TB risk diseases.

## 2. Materials and methods

### 2.1. Study population

In 2017 and 2018, individuals from eight congregate settings underwent LTBI screening with either interferon gamma release assay (IGRA) or tuberculin skin test (TST). The number of source population and participation rate among them were described in our previous article (9). Candidates for military conscription were not included in this study, as the informed consent of participants with negative IGRA results was not collected by Military Manpower Administration of South Korea, thus analysis of LTBI prevalence was unfeasible. Exclusion criteria were: (1) participants who were not registered with National Health Insurance of South Korea as the linkage with National Health Information Database (NHID) was unfeasible for these people; (2) those with missing data (e.g., date of LTBI examination); (3) participants who underwent only TST due to concern of possible high false positive rate in TST results (10) considering high Bacillus Calmette–Guérin vaccination rate in South Korea (11); (4) participants who had previous records of TB notification in Korean National TB Surveillance System (KNTSS) before the date of LTBI screening; and (5) participants who initiated LTBI treatment as TB contacts before the date of LTBI screening as they were not valid targets for LTBI screening. However, those were included in the analysis of past TB exposure history and medical risk group.

### 2.2. Data linkage and study design

Study design, LTBI screening process and database which constitute our cohort and methods for data linkage were describe in a previous protocol article (12). Participants underwent IGRA with QuantiFERON-TB Gold In-Tube tests (Qiagen, Hilden, Germany). Results were interpreted according to the manufacturer's manual. Participants with positive IGRA test were recommended to visit a public health center or a private hospital for further examination of active TB with chest X-ray and sputum study, if needed. Korea Disease Control and Prevention Agency collected information of age, sex, types of congregate setting, types of occupation, results of IGRA, and chest X-ray. This database was linked with NHID including information on comorbidities, income level, and home address (district, city or county level) and KNTSS consisting of TB notification records of the participants, with joint keys which anonymized personal identification number assigned to each South Korean population by the government. Additionally, databases of TB contact investigation in congregate settings and household contacts which was fully implemented in 2013 was linked in the same way (8).

In this cross-sectional study, the prevalence of LTBI among participants and risk factors for LTBI were investigated. All participants were classified into three groups based on the purpose of LTBI screening (9). Participants whose purpose of LTBI screening was to reduce secondary TB cases in young generation (workers of postpartum care centers, social welfare facilities and educational institutions such as daycare centers, kindergartens, elementary and secondary schools) were assigned to Group A. Participants of young generation (first year students in high school and out-of-school youths) were assigned into Group B. Inmates of correctional facilities were assigned into Group C. In addition, prevalence of concurrent active TB, which was defined as TB cases notified within 30 days from the date of LTBI test, was calculated (12).

In Korean guidelines for tuberculosis, contacts and patients with high TB risk diseases are two main target groups for LTBI screening and treatment (13). To elucidate how many IGRA-positive participants could be identified with the conventional LTBI strategy targeting only contacts and medical high-risk groups especially in young generation aged under 35, past TB exposure history since 2013 and underlying diseases of participants which could increase the risk for TB were investigated using national contact investigation database and NHID, respectively. To identify the proportion of participants with past TB exposure, those who underwent LTBI treatment previously in the process of contact investigation were included in this analysis. Past exposure history was classified as exposure that occurred within 2 years from the date of LTBI screening and that occurred beyond 2 years. All contacts underwent chest X-ray. LTBI test (TST or IGRA) was recommended only for close contacts (14). Participants with past exposure history were categorized based on results of LTBI test of contact investigation. Comorbidities of participants such as high TB risk diseases [HIV infection, post-organ transplantation status, anti-tumor necrosis factor (TNF) treatment) and moderate TB risk diseases (end-stage renal disease (ESRD), post-gastrectomy status, head and neck cancer, hematologic malignancy, and diabetes mellitus (DM)] at the timepoint of the LTBI screening date were extracted from NHID. Proportions of participants with positive IGRA results which could be covered by the strategy targeting (1) only high-risk group (Strategy 1), (2) up to moderate-risk group except DM (Strategy 2), and (3) up to moderate risk group including DM (Strategy 3) were calculated to estimate the coverage proportion of the current national LTBI strategy.

### 2.3. Exposure variables

In this study, five age groups were defined. Participants aged below 20 years who were mostly young adolescents and those who aged 65 years or more (elderly population) were classified. Other age groups (20–64 years) were categorized with the same interval of 15 years – those aged 20–34 years, 35–49 years, and 50–64 years, respectively. Considering that this LTBI screening program targets workers in each congregate setting, participants aged below 20 years were rare except for Group B. Therefore, those aged below 35 years were set as a reference age group among Groups A and C.

Place of residence was classified based on municipal level administrative divisions – metropolitan city (district), small to medium-sized city (city), or rural area (county). Income level which was annually investigated by National Health Insurance Service was determined with wage income and value of property such as houses and vehicles (15). Income level was presented with quartiles – low, moderate-low, moderate-high, and high. Participants' comorbidities were described with Charlson comorbidity index (CCI) calculated with International Classification of Diseases, Tenth Revision (ICD-10) codes (16). Participants were divided into four groups – those with CCI score 0, those with score 1, those with score 2, and those with score 3 or more.

### 2.4. Statistical analysis

To identify risk factors for LTBI, multivariable Poisson regression with a robust variance estimator was used to estimate the relative risk. Participants with missing values of income level or place of residence were excluded from the multivariable analysis. All statistical analyses were conducted with R v.3.6.2 (R foundation for Statistical Computing, Vienna, Austria) and SAS software version 9.4 (SAS Institute Inc., Cary, NC, USA).

### 2.5. Ethical approval

The present study protocol was reviewed and approved by the Institutional Review Board (IRB) of Incheon St. Mary's Hospital, the Catholic University of Korea (IRB No. OC19ZESE0023). Korea Disease Control and Prevention Agency collected informed consent from all participants when they were enrolled according to Tuberculosis Prevention Act. The study was conducted in accordance with the Declaration of Helsinki.

## 3. Results

**Figure 1:**
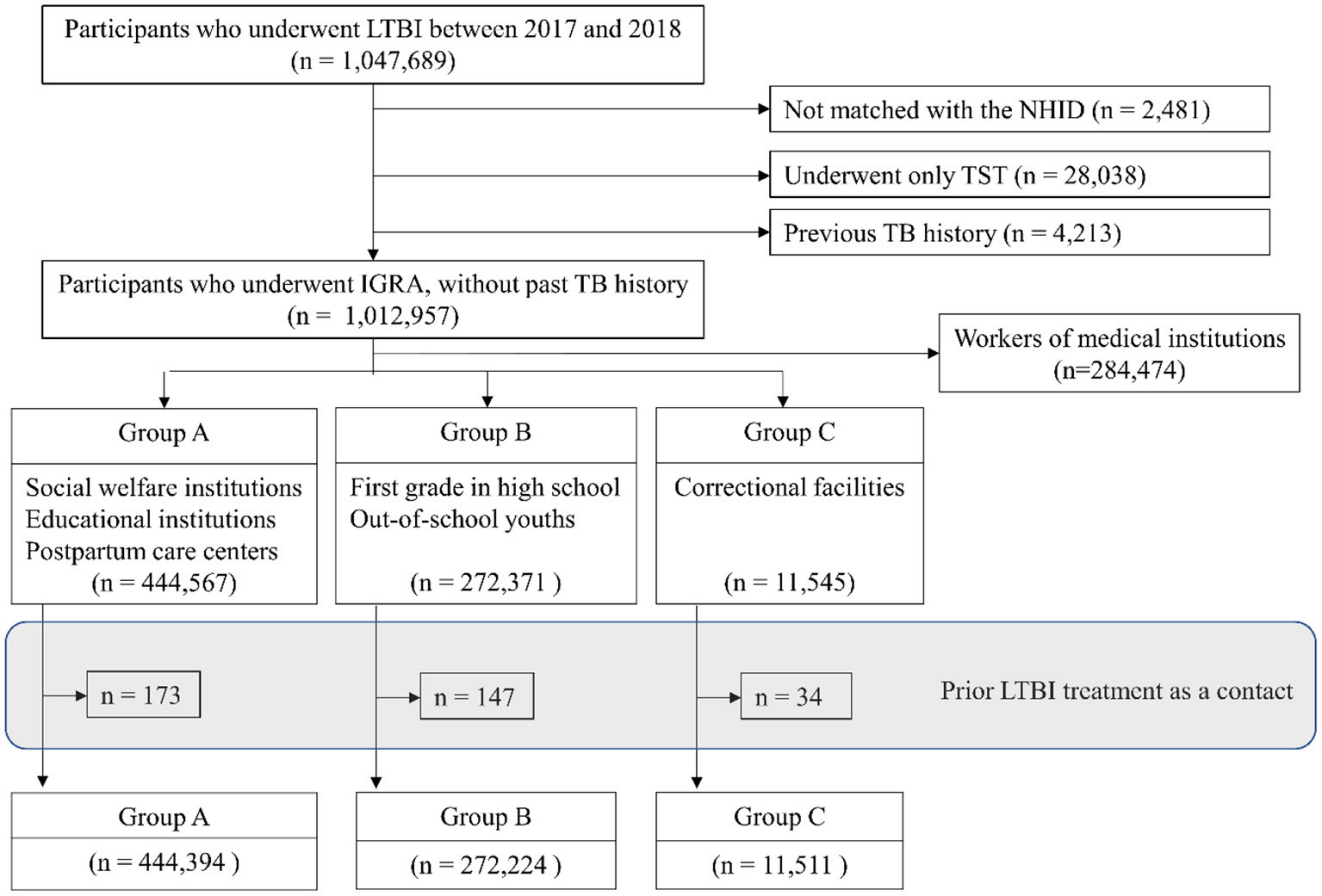
Flow diagram. LTBI, latent tuberculosis infection; NHID, National Health Insurance Database; TST, tuberculin skin test; TB, tuberculosis; IGRA, interferon-gamma release assay. Group A: workers of postpartum care centers, social welfare facilities and educational institutions, Group B: first year students in high school and out-of-school youths, Group C: inmates of correctional facilities.

A total of 444,394 participants in Group A, 272,224 participants in Group B, and 11,511 participants in Group C were included in this study (Figure 1). Numbers of concurrent active TB patients were 23, 9, and 0 in Group A, Group B, and Group C, respectively. Prevalence by each variable is presented in Table 1.

**Table 1:** Prevalence of concurrent active TB among the total participants in each group.

	**Group A**			**Group B**			**Group C**		
	**TB (*n*)**	**Total (*n*)**	**Prevalence (/100,000 population)**	**TB (*n*)**	**Total (*n*)**	**Prevalence (/100,000 population)**	**TB (*n*)**	**Total (*n*)**	**Prevalence (/100,000 population)**
Total	23	444,394	5.2	9	272,224	3.3	0	11,511	0
**Gender**									
Male	5	36,169	13.8	5	151,523	3.3	0	10,798	0
Female	18	408,225	4.4	4	120,701	3.3	0	713	0
**Age, years**									
<20	0	0	**-**	9	272,224	3.3	0	0	**-**
20-34	5	121,522	4.1	0	0	**-**	0	2,803	0
35–49	10	204,152	4.9	0	0	**-**	0	4,759	0
50–64	7	108,876	6.4	0	0	**-**	0	3,536	0
≥ 65	1	9,844	10.2	0	0	**-**	0	413	0
**Place of residence** ^a^									
Metropolitan city	11	247,920	4.4	5	161,379	3.1	0	6,093	0
Small to medium-sized city	7	154,516	4.5	1	84,530	1.2	0	3,324	0
Rural area	5	41,864	11.9	3	26,099	11.5	0	994	0
**Income level** ^b^									
Low	7	199,127	3.5	1	58,063	1.7	0	5,406	0
Moderate low	10	133,779	7.5	0	39,543	0.0	0	2,150	0
Moderate high	4	65,385	6.1	2	54,939	3.6	0	1,555	0
High	2	40,041	5.0	6	115,287	5.2	0	1,529	0
**Charlson comorbidity index**									
Score 0	9	209,149	4.3	7	192,920	3.6	0	7,889	0
Score 1	8	151,653	5.3	1	69,772	1.4	0	2,200	0
Score 2	4	56,729	7.1	1	8,582	11.7	0	850	0
Score 3 or more	2	26,863	7.4	0	950	0.0	0	572	0

a Number of missing value was 1,410.

b Number of missing value was 11,325. Group A: workers of postpartum care centers, social welfare facilities and educational institutions, Group B: first year students in high school and out-of-school youths, Group C: inmates of correctional facilities.

### 3.1. Prevalence of LTBI

Overall prevalence of LTBI in Group A, Group B, Group C were 20.7, 2.0, and 33.2%, respectively. In Group A, prevalence in males (28.4%) was higher than that in females (20.0%) (Table 2). Prevalence increased with increasing age. It was 6.6% in young adults (age < 35 years) and 44.1% in the elderly population (age ≥ 65 years). Participants with more comorbidities (CCI score ≥ 3) showed higher prevalence (30.2%) than those without comorbidities (CCI score 0) (18.5%).

**Table 2:** Prevalence of LTBI in Group A (workers of postpartum care centers, social welfare facilities and educational institutions) and risk factors for LTBI.

**Variables**	**IGRA positive (row %)**	**IGRA negative (row %)**	**IGRA indeterminate (row %)**	**Total**	**Univariable analysis**		**Multivariable analysis**	
					**RR (95% CI)**	***p*–value**	**aRR (95% CI)**	***p*–value**
Participants, No.	92,048 (20.7)	352,062 (79.2)	284 (0.1)	444,394				
**Gender**								
Male	10,258 (28.4)	25,877 (71.5)	34 (0.1)	36,169	1.41 (1.38–1.43)	<0.001	1.41 (1.38–1.43)	<0.001
Female	81,790 (20.0)	32,6185 (79.9)	250 (0.1)	408,225	1		1	
**Age, years**				
<35	8,026 (6.6)	113,447 (93.4)	49 (0.0)	121,522	1		1	
35–49	40,607 (19.9)	163,434 (80.1)	111 (0.1)	204,152	3.03 (2.96–3.10)	<0.001	3.03 (2.96–3.10)	<0.001
50–64	39,072 (35.9)	69,699 (64.0)	105 (0.1)	108,876	5.46 (5.33–5.58)	<0.001	5.46 (5.33–5.58)	<0.001
≥ 65	4,343 (44.1)	5,482 (55.7)	19 (0.2)	9,844	6.70 (6.49–6.91)	<0.001	6.70 (6.49–6.91)	<0.001
**Place of residence** ^a^				
Metropolitan city	51,135 (20.6)	196,628 (79.3)	157 (0.1)	247,920	0.97 (0.95–0.99)	<0.001	0.97 (0.95–0.99)	<0.001
Small to medium-sized city	31,949 (20.7)	122,473 (79.3)	94 (0.1)	154,516	0.97 (0.95–0.99)	0.004	0.97 (0.95–0.99)	0.004
Rural area	8,928 (21.3)	32,903 (78.6)	33 (0.1)	41,864	1		1	
**Income level** ^b^								
Low	42,689 (21.4)	156,310 (78.5)	128 (0.1)	199,127	1		1	
Moderate low	25,938 (19.4)	107,763 (80.6)	78 (0.1)	133,779	0.90 (0.89–0.92)	<0.001	0.90 (0.89–0.92)	<0.001
Moderate high	13,330 (20.4)	52,016 (79.6)	39 (0.1)	65,385	0.95 (0.94–0.97)	<0.001	0.95 (0.94–0.97)	<0.001
High	9,166 (22.9)	30,843 (77.0)	32 (0.1)	40,041	1.07 (1.05–1.09)	<0.001	1.07 (1.05–1.09)	<0.001
**Charlson comorbidity index**								
Score 0	38,682 (18.5)	170,364 (81.5)	103 (0.0)	209,149	1		1	
Score 1	31,136 (20.5)	120,415 (79.4)	102 (0.1)	151,653	1.11 (1.10–1.13)	<0.001	1.11 (1.10–1.13)	<0.001
Score 2	14,116 (24.9)	42,562 (75.0)	51 (0.1)	56,729	1.34 (1.32–1.37)	<0.001	1.34 (1.32–1.37)	<0.001
Score 3 or more	8,114 (30.2)	18,721 (69.7)	28 (0.1)	26,863	1.63 (1.60–1.66)	<0.001	1.63 (1.60–1.66)	<0.001

Data were expressed as number and row percentage.

a Number of missing value was 94.

b Number of missing value was 6,062. IGRA, interferon-gamma release assay; RR, relative risk; aRR, adjusted relative risk.

In Group B, prevalence in males (2.0%) was similar to that in females (2.0%) (Table 3). Prevalence decreased with higher income level. It was 1.8% in the high-income group and 2.4% in the low-income group. In group C, the prevalence in males (33.7%) was higher than that in females (24.8%), elderly population (age ≥ 65 years) (55.0%) than in young adults (age < 35 years) (13.1%), and participants with more comorbidities (CCI score ≥ 3) (46.3%) than in those without comorbidities (CCI score 0) (31.9%) (Table 4). The prevalence was 27.9% in the high-income group and 35.9% in the low-income group.

**Table 3:** Prevalence of LTBI in Group B (first year students in high school and out-of-school youths) and risk factors for LTBI.

**Variables**	**IGRA positive (row %)**	**IGRA negative (row %)**	**IGRA indeterminate (row %)**	**Total**	**Univariable analysis**		**Multivariable analysis**	
					**RR (95% CI)**	***p*–value**	**aRR (95% CI)**	***p*–value**
Participants, No.	5,508 (2.0)	266,643 (97.9)	73 (0.0)	272,224				
**Gender**								
Male	3,049 (2.0)	148,428 (98.0)	46 (0.0)	151,523	0.98 (0.93–1.04)	0.544	0.98 (0.93–1.04)	0.544
Female	2,459 (2.0)	118,215 (97.9)	27 (0.0)	120,701	1		1	
**Place of residence** ^a^								
Metropolitan city	3,248 (2.0)	158,079 (98.0)	52 (0.0)	161,379	0.94 (0.86–1.03)	0.186	0.94 (0.86–1.03)	0.186
Small to medium-sized city	1,693 (2.0)	82,820 (98.0)	17 (0.0)	84,530	0.93 (0.85–1.02)	0.143	0.93 (0.85–1.02)	0.143
Rural area	565 (2.2)	25,530 (97.8)	4 (0.0)	26,099	1		1	
**Income level** ^b^								
Low	1,381 (2.4)	56,659 (97.6)	23 (0.0)	58,063	1		1	
Moderate low	877 (2.2)	38,660 (97.8)	6 (0.0)	39,543	0.93 (0.86–1.01)	0.095	0.93 (0.86–1.01)	0.095
Moderate high	1111 (2.0)	53,814 (98.0)	14 (0.0)	54,939	0.85 (0.79–0.92)	<0.001	0.85 (0.79–0.92)	<0.001
High	2,051 (1.8)	113,206 (98.2)	30 (0.0)	115,287	0.75 (0.70–0.80)	<0.001	0.75 (0.70–0.80)	<0.001
**Charlson comorbidity index**								
Score 0	3859 (2.0)	189,010 (98.0)	51 (0.0)	192,920	1		1	
Score 1	1440 (2.1)	68,312 (97.9)	20 (0.0)	69,772	1.03 (0.97–1.10)	0.325	1.03 (0.97–1.10)	0.325
Score 2	177 (2.1)	8,403 (97.9)	2 (0.0)	8,582	1.03 (0.89–1.20)	0.665	1.03 (0.89–1.20)	0.665
Score 3 or more	32 (3.4)	918 (96.6)	0 (0.0)	950	1.60 (1.12–2.28)	0.009	1.60 (1.12–2.28)	0.009

Data were expressed as number and row percentage.

a Number of missing value was 216.

b Number of missing value was 4,392. IGRA, interferon-gamma release assay; RR, relative risk; aRR, adjusted relative risk.

**Table 4:** Prevalence of LTBI in Group C (inmates of correctional facilities) and risk factors for LTBI.

**Variables**	**IGRA positive (row %)**	**IGRA negative (row %)**	**IGRA indeterminate (row %)**	**Total**	**Univariable analysis**		**Multivariable analysis**	
					**RR (95% CI)**	***p*-value**	**aRR (95% CI)**	***p*-value**
Participants, No.	3,817 (33.2)	7,683 (66.7)	11 (0.1)	11511				
**Gender**								
Male	3,640 (33.7)	7,147 (66.2)	11 (0.1)	10798	1.36 (1.19-1.57)	<0.001	1.46 (1.27-1.67)	<0.001
Female	177 (24.8)	536 (75.2)	0 (0.0)	713	1		1	
**Age, years**								
<35	368 (13.1)	2,432 (86.8)	3 (0.1)	2,803	1		1	
35 – 49	1,436 (30.2)	3,318 (69.7)	5 (0.1)	4,759	2.37 (2.12-2.65)	<0.001	2.38 (2.13-2.67)	<0.001
50 – 64	1,786 (50.5)	1,748 (49.4)	2 (0.1)	3,536	3.96 (3.55-4.41)	<0.001	3.95 (3.54-4.40)	<0.001
≥ 65	227 (55.0)	185 (44.8)	1 (0.2)	413	4.33 (3.77-4.97)	<0.001	4.33 (3.77-4.98)	<0.001
**Place of residence** ^a^								
Metropolitan city	2,016 (33.1)	4,073 (66.8)	4 (0.1)	6,093	0.96 (0.87-1.05)	0.342	0.99 (0.91-1.09)	0.871
Small to medium-sized city	1,031 (31.0)	2,291 (68.9)	2 (0.1)	3,324	0.89 (0.80-0.98)	0.019	0.94 (0.85-1.03)	0.204
Rural area	345 (34.7)	646 (65.0)	3 (0.3)	994	1		1	
**Income level** ^b^								
Low	1,942 (35.9)	3,458 (64.0)	6 (0.1)	5,406	1		1	
Moderate low	734 (34.1)	1,414 (65.8)	2 (0.1)	2,150	0.94 (0.87-1.01)	0.084	1.00 (0.93-1.07)	0.878
Moderate high	401 (25.8)	1,154 (74.2)	0 (0.0)	1,555	0.72 (0.66-0.79)	<0.001	0.87 (0.80-0.95)	0.002
High	427 (27.9)	1,101 (72.0)	1 (0.1)	1,529	0.78 (0.72-0.85)	<0.001	0.75 (0.69-0.82)	<0.001
**Charlson comorbidity index**								
Score 0	2,520 (31.9)	5,362 (68.0)	7 (0.1)	7,889	1		1	
Score 1	709 (32.2)	1,488 (67.6)	3 (0.1)	2,200	1.03 (0.96-1.10)	0.479	0.99 (0.93-1.07)	0.874
Score 2	323 (38.0)	527 (62.0)	0 (0.0)	850	1.20 (1.09-1.32)	<0.001	1.00 (0.91-1.10)	0.967
Score 3 or more	265 (46.3)	306 (53.5)	1 (0.2)	572	1.51 (1.37-1.66)	<0.001	1.10 (1.00-1.21)	0.051

Data were expressed as number and row percentage.

a Number of missing value was 1,100.

b Number of missing value was 871. IGRA, interferon-gamma release assay; RR, relative risk; aRR, adjusted relative risk.

### 3.2. Risk factors for LTBI

The most important variable that affected the prevalence in Group A was age (Table 2). Compared with young adults (age < 35 years), adjusted relative risks (aRRs) in participants aged 35–49 years, those aged 50–64 years, and those aged 65 years or more were 3.06 [95% confidence interval (CI): 2.99–3.13), 5.41 (95% CI: 5.29–5.54), and 6.36 (95% CI: 6.16–6.57], respectively. Compared with results of univariable analysis, after adjusting for other variables including age, effects of place of residence were reversed and those of comorbidities were decreased. The trend of higher LTBI prevalence with lower income level was more prominent in multivariable analysis than in univariable analysis.

In Group B, which was composed of people with same age and thus effect of age as a confounder was excluded, effect of income level was significant (Table 3). Compared with the low-income group, aRRs of moderate-low, moderate-high, and high-income groups were 0.93 (95% CI: 0.86–1.01), 0.85 (95% CI: 0.79–0.92), and 0.75 (95% CI: 0.70–0.80), respectively. Adolescents with more comorbidities (CCI score ≥ 3) showed significantly higher risk for LTBI (aRR: 1.56, 95% CI: 1.10–2.22).

Group C and Group A showed similar trends, both showing a profound impact of age on LTBI prevalence (Table 4). Effects of gender and income level were more prominent in Group C than in Group A. However, the trend of the relationship between participants with more comorbidities and higher risk of LTBI was obscure in Group C.

### 3.3. Coverage of current LTBI strategy among participants

Past TB exposure history between 2013 and the date of LTBI screening among participants with positive IGRA results is presented in Table 5. Proportions of participants who had past exposure history were 2.7, 4.4, and 3.3% in Group A, Group B, and Group C, respectively. Proportions of recent exposure defined as an exposure within 2 years before the screening date were 1.5, 2.4, and 1.8% in Group A, Group B, and Group C, respectively. When participants with positive IGRA results who aged below 35 years were analyzed, proportions of participants who had past exposure history were 2.0 and 3.3% in Group A and Group C, respectively.

**Table 5:** Coverage rate of current LTBI strategy targeting for TB contacts among the participants with positive IGRA result in each group.

	**Group A, IGRA (**+**)**		**Group B, IGRA (+)**	**Group C, IGRA (**+**)**	
	**All ages**	**Age < 35**	**All ages (Age < 35)**	**All ages**	**Age < 35**
Total	92,168 (100.0)	8,044 (100.0)	5,554 (100.0)	3,846 (100.0)	370 (100.0)
1. No known past TB exposure	89,658 (97.3)	7,886 (98.0)	5,308 (95.6)	3,720 (96.7)	356 (96.2)
2. Known past TB exposure	2,510 (2.7)	158 (2.0)	246 (4.4)	126 (3.3)	14 (3.8)
2A. Past TB exposure less than two years before the screening	1,420 (1.5)	57 (0.7)	133 (2.4)	70 (1.8)	4 (1.1)
2A-1. No LTBI test	1,105 (1.2)	34 (0.4)	70 (1.3)	39 (1.0)	4 (1.1)
2A-2. Not LTBI	76 (0.1)	9 (0.1)	30 (0.5)	2 (0.1)	0 (0.0)
2A-3. LTBI	238 (0.3)	14 (0.2)	33 (0.6)	29 (0.8)	0 (0.0)
2A-4. Indeterminate	1 (0.0)	0 (0.0)	0 (0.0)	0 (0.0)	0 (0.0)
2B. Past TB exposure more than two years before the screening	1,090 (1.2)	101 (1.3)	113 (2.0)	56 (1.5)	10 (2.7)
2B-1. No LTBI test	493 (0.5)	22 (0.3)	10 (0.2)	15 (0.4)	2 (0.5)
2B-2. Not LTBI	145 (0.2)	29 (0.4)	36 (0.6)	5 (0.1)	1 (0.3)
2B-3. LTBI	452 (0.5)	50 (0.6)	67 (1.2)	36 (0.9)	7 (1.9)
2B-4. Indeterminate	0 (0.0)	0 (0.0)	0 (0.0)	0 (0.0)	0 (0.0)

LTBI, latent tuberculosis infection; TB, tuberculosis; IGRA, interferon-gamma release assay. Group A: workers of postpartum care centers, social welfare facilities and educational institutions, Group B: first year students in high school and out-of-school youths, Group C: inmates of correctional facilities.

Among participants with positive IGRA results in each group, proportions of participants with high or moderate TB risk diseases specified in the current LTBI guideline of South Korea are presented in Table 6. High TB risk diseases accounted for only up to 0.2% of total participants with positive IGRA results in each group (Strategy 1). In addition, strategy additionally targeting moderate TB risk diseases (except for DM, Strategy 2) and high TB risk diseases covered only 1.0, and 1.7% of participants with positive IGRA results in Group A and Group C, respectively. The strategy including DM (Strategy 3) covered 6.5, 0.6 and 12.5% of participants with positive IGRA results in Groups A, B, and C, respectively.

**Table 6:** Coverage rate of current LTBI strategy targeting for high or moderate TB risk diseases among the participants with positive IGRA result in each group.

	**Group A, IGRA (**+**)**		**Group B, IGRA (+)**	**Group C, IGRA (**+**)**	
	**All ages**	**Age < 35**	**All ages (Age < 35)**	**All ages**	**Age < 35**
Total	92,168 (100.0)	8,044 (100.0)	5,554 (100.0)	3,846 (100.0)	370 (100.0)
**High TB risk diseases**					
- HIV infection	8 (0.0)	0 (0.0)	1 (0.0)	1 (0.0)	0 (0.0)
- Post-organ transplantation status	49 (0.1)	4 (0.0)	4 (0.1)	5 (0.1)	0 (0.0)
- Anti-TNF treatment	43 (0.0)	4 (0.0)	4 (0.1)	0 (0.0)	0 (0.0)
**Moderate TB risk diseases**					
- ESRD	81 (0.1)	3 (0.0)	1 (0.0)	26 (0.7)	2 (0.5)
- Post-gastrectomy status	274 (0.3)	3 (0.0)	0 (0.0)	18 (0.5)	0 (0.0)
- Head and neck cancer	284 (0.3)	3 (0.0)	2 (0.0)	15 (0.4)	0 (0.0)
- Hematologic malignancy	183 (0.2)	13 (0.2)	9 (0.2)	4 (0.1)	0 (0.0)
- DM	5,190 (5.6)	73 (0.9)	15 (0.3)	433 (11.3)	2 (0.5)
Strategy 1^a^	100 (0.1)	8 (0.1)	9 (0.2)	6 (0.2)	0 (0.0)
Strategy 2	877 (1.0)	25 (0.3)	19 (0.3)	65 (1.7)	2 (0.5)
Strategy 3	5,966 (6.5)	97 (1.2)	34 (0.6)	479 (12.5)	4 (1.1)

LTBI, latent tuberculosis infection; TB, tuberculosis; IGRA, interferon-gamma release assay; HIV, human immunodeficiency virus; TNF, tumor necrosis factor; ESRD, end-stage renal disease; DM, diabetes mellitus. Group A: workers of postpartum care centers, social welfare facilities and educational institutions, Group B: first year students in high school and out-of-school youths, Group C: inmates of correctional facilities.

a Strategy 1 covers for only high TB risk diseases, Strategy 2 for high or moderate TB risk diseases except for DM, and Strategy for high or moderate TB risk diseases including DM.

## 4. Discussion

In this study, we investigated the prevalence of LTBI and its risk factors among participants of the national LTBI screening program in South Korea. Male, old age, low-income level, and higher comorbidity index were risk factors for LTBI. Current LTBI strategy in South Korea could cover only a small proportion of the current LTBI reservoir, which underscored the necessity of expanding the LTBI target group.

Age was the single most important risk factor for LTBI which overwhelmed effects of other variables. This finding reflects the large generation gap in TB infection in South Korea currently. Intuitively, elderly people who were born before 1950s experienced the Korean War (1950–1953) in their childhood. After the war, they were exposed to community's high TB burden during the period of rapid economic growth until the 1980s. Low accessibility to medical service and many cases of incomplete treatment due to suboptimal regimen or absence of support for treatment adherence during that period led to the current large pool of LTBI among the elderly population. In contrast, young population born after 1980s were exposed to relatively low TB burden as a result of marked decrease in TB burden until 2000 (7). The introduction of a universal population coverage of National Health Insurance in 1989 enhanced the accessibility to medical service. The full use of rifampicin since 1980 has enabled a short-course therapy with successful outcome, which contributes to a decrease in community's TB burden (11).

Such a large generation gap in LTBI prevalence could be explained by the age-period-cohort effect. However, estimation of the exact extent of each effect was unfeasible as this survey was performed at a single timepoint. Further regular population based LTBI surveys based on IGRA are needed to estimate each effect. Moreover, considering that South Korea is a rapidly aging country, accurate prediction for national TB burden in the future considering the impact of population aging could be feasible by estimating such effects (17).

Interestingly, age effect in the age-period-cohort model was not so prominent when LTBI was diagnosed by IGRA, not by TST. In several previous studies, the rate of TST positivity in elderly population is decreased possibly due to waning immunity (18, 19). Similarly, in the 7th Korea National Health and Nutrition Examination Survey, the prevalence of LTBI in the general population in South Korea increased with increasing age, culminating in people in their 50s (48.7%) and slightly decreasing in people in their 60s (45.0%) (20). In the elderly population, decrease of delayed type hypersensitivity reaction might have caused false-negative TST results and underestimation of LTBI prevalence. However, unlike TST, it is known that age effect is not so prominent in IGRA. Our results revealed that elderly people in South Korea who were exposed to the highest nationwide TB burden since the Korean War showed the highest relative risk for LTBI.

Aging has a significant impact on TB burden in the future, which hampers the decline in TB incidence due to a high risk of endogenous TB reactivation in the elderly population (17, 21). Our study demonstrates a large LTBI reservoir in the elderly population currently in South Korea. Although the importance of tackling LTBI has been underscored for TB elimination (3), screening and treating LTBI in the general elderly population are currently not recommended due to low cost-effectiveness attributable to low predictive values of current diagnostic tools for LTBI and higher frequency of adverse events during LTBI treatment in the elderly population (22, 23). However, Huynh et al. have demonstrated that China could achieve global target of 90% reduction in TB incidence and 95% reduction in TB mortality by 2035 when the preventive therapy for elderly population is added to other interventions (24). Recently, expanding LTBI treatment to the elderly population has been suggested (25, 26), especially in intermediate TB burden countries where control of a large LTBI reservoir in the elderly population is a key strategy for reducing national TB burden. In Taiwan, successful LTBI treatment for elderly patients with poorly controlled DM has been reported (27). Further studies investigating the feasibility of LTBI treatment among elderly population is needed.

In several previous studies, low-income level is a risk factor for LTBI (28, 29). However, another study showed no association between income level and LTBI prevalence in the general population in Singapore (30). We demonstrated that lower income was associated with higher LTBI prevalence in a large-scaled general population. Especially, among first-year students in high school, which was composed of more than half of the total nationwide population born in 1 year (9), that trend was obvious. In addition, as we used individual income level collected by National Health Insurance Service to impose a personal health insurance premium, we expect our data are more accurate than self-reported income levels in previous studies (28, 30). Considering that TB incidence is relatively high among the population with a low socioeconomic status (31), we intuitively speculate that there might be more risk of household TB exposure in participants with low-income level. However, other factors such as DM, which is more prevalent in the low-income group than in the high income group (32), might have contributed to the high LTBI prevalence in the low-income group as demonstrated in previous studies (33, 34).

TB in incarcerated population has been a public health issue, globally (35). In a previous study comparing the prevalence of several diseases among prisoners and that among general population in South Korea, standardized prevalence ratio of pulmonary TB was 9.58, which demonstrated that prisoners were vulnerable population (36). In previous studies investigating LTBI status in incarcerated population, 10 out of 1,422 prisoners had concurrent active TB diseases in Brazil (37), and 2 out of 1,208 in Iran (38). However, in our study, there was no prevalent TB cases identified during LTBI screening. This might result from the effect of annual medical checkup including chest X-ray for prisoners in South Korea (39), which enabled exclusion of prisoners with active TB diseases from this analysis. Indeed, 134 participants in Group C (11.5 per 1,000 participants) with previous TB history were excluded in this analysis, which was a higher proportion than that in Group A (1,956 participants, 4.4 per 1,000 participants) and Group B (110 participants, 0.4 per 1,000 participants). In addition, as we defined prevalent TB case as that notified within 30 days from the date of LTBI examination (12), we speculate that 30 days would be insufficient for diagnosis of TB in current setting of correctional facilities, as prisoner's access to healthcare services is limited and the diagnosis is often delayed (36). Therefore, incidence of TB in correctional facilities by LTBI status of each prisoner should be investigated, which would demonstrate the TB burden in correctional facilities better. Additionally, further studies covering the time delays in diagnosis of TB in correctional facilities are needed.

To investigate how many new TB infection could be covered by contact investigation in the young generation, we calculated annual risk of TB infection in Group B with a simplified method using prevalence (2.08%) and mean age (17 years old) (40). In that way, the number of new TB infection in one-year was ~119.82 cases per 100,000 population. This figure might have been underestimated and represents minimal annual risk of infection considering results of a previous study demonstrating that the risk of TB infection is increased from birth to 20 years of age (41). However, through contact investigation, 20.93 cases of new TB infection per 100,000 population (114 cases among 272,371 total participants within recent 2 years) were identified annually (15.97 cases by contact investigation in congregate settings and 4.96 cases by household contact investigation) (Table 7). Therefore, contact investigation covers only a limited proportion of new TB infections in young generation. Similarly, a previous study carried out in Uganda has demonstrated that unrecognized exposure to infectious cases in the community is important in the transmission of TB infection, which occurs outside the net of contact investigation (42). In addition, with the national LTBI strategy targeting patients with high TB risk diseases, only up to 0.6% of LTBI cases could be covered among young generation when patients with DM are included in target group. Thus, the current national LTBI strategy is not enough for decreasing LTBI reservoir in the young generation who showed the highest risk of progression into TB disease among all age groups (22). Similar to our findings, application of WHO's recommendation for LTBI screening minimally impacted TB incidence in one Canadian province (43). However, mass screening strategy with IGRA, as in Group A and Group B of this study, would inevitably lead to low positive predictive value (PPV) for TB which might impair cost-effectiveness. Therefore, further high-risk group should be identified along with the introduction of new biomarkers for predicting TB development (44) which can enhance PPV.

**Table 7:** Coverage rate of current LTBI strategy targeting for TB contacts (overall/contacts in congregate settings/household contacts) among the total participants in each group.

	**Group A, total participants**			**Group B, total participants**			**Group C, total participants**		
	**Overall**	**Contacts in Congregate setting**	**Household contacts**	**Overall**	**Contacts in Congregate setting**	**Household contacts**	**Overall**	**Contacts in Congregate setting**	**Household contacts**
Total	444,567 (100)	444,567 (100)	444,567 (100)	272,371 (100)	272,371 (100)	272,371 (100)	11,545 (100)	11,545 (100)	11,545 (100)
1. No known past TB exposure	434,325 (97.7)	435,748 (98.0)	443.100 (99.7)	265.515 (97.5)	266.397 (97.8)	271,462 (99.7)	11,197 (97.0)	11,215 (97.1)	11,527 (99.8)
2. Known past TB exposure	10,242 (2.3)	8,819 (2)	1,467 (0.3)	6856 (2.5)	5,974 (2.2)	909 (0.3)	348 (3)	330 (2.9)	18 (0.2)
2A. Past TB exposure less than two years before the screening	5,209 (1.2)	4,706 (1.1)	519 (0.1)	4541 (1.7)	4,278 (1.6)	280 (0.1)	191 (1.7)	187 (1.6)	4 (0.0)
2A-1. No LTBI test	3,603 (0.8)	3,314 (0.7)	302 (0.1)	3015 (1.1)	3,003 (1.1)	26 (0.0)	101 (0.9)	97 (0.8)	4 (0.0)
2A-2. Not LTBI	1,264 (0.3)	1,085 (0.2)	181 (0.0)	1411 (0.5)	1,187 (0.4)	227 (0.1)	55 (0.5)	55 (0.5)	0 (0)
2A-3. LTBI	340 (0.1)	305 (0.1)	36 (0.0)	114 (0.0)	87 (0.0)	27 (0)	35 (0.3)	35 (0.3)	0 (0)
2A-4. Indeterminate	2 (0.0)	2 (0.0)	0 (0)	1 (0.0)	1 (0.0)	0 (0)	0 (0)	0 (0)	0 (0)
2B. Past TB exposure more than two years before the screening	5,033 (1.1)	4,113 (0.9)	948 (0.2)	2,315 (0.8)	1,696 (0.6)	629 (0.2)	157 (1.4)	143 (1.2)	14 (0.1)
2B-1. No LTBI test	1,625 (0.4)	1,231 (0.3)	413 (0.1)	162 (0.1)	69 (0.0)	96 (0.0)	32 (0.3)	28 (0.2)	4 (0.0)
2B-2. Not LTBI	2,263 (0.5)	1,906 (0.4)	366 (0.1)	1,799 (0.7)	1,399 (0.5)	406 (0.1)	58 (0.5)	49 (0.4)	9 (0.1)
2B-3. LTBI	1,144 (0.3)	976 (0.2)	168 (0.0)	354 (0.1)	228 (0.1)	127 (0.0)	67 (0.6)	66 (0.6)	1 (0.0)
2B-4. Indeterminate	1 (0.0)	0 (0)	1 (0)	0 (0)	0 (0)	0 (0)	0 (0)	0 (0)	0 (0)

Data were expressed as number and columnar percentage. LTBI, latent tuberculosis infection; TB, tuberculosis. Group A: workers of postpartum care centers, social welfare facilities and educational institutions, Group B: first year students in high school and out-of-school youths, Group C: inmates of correctional facilities.

Our study performed an unprecedented large-scaled LTBI survey using IGRA. We linked survey data to national contact investigation database and NHID covering the entire South Korean population which enhanced data integrity. However, our study has several limitations. First, only half of source population participated in this screening program, which could be potential source of selection bias (9). Second, there were participants with missing values of income levels or places of residence, especially among inmates of correctional facilities who lost qualification of National Health Insurance. Third, due to the cross-sectional design of this study, investigating the incidence of TB infection, which might be presented with positive conversion of IGRA, was unfeasible.

## 5. Conclusion

In conclusion, among participants for national LTBI screening program in South Korea, old age and low-income level were associated with LTBI. Only a small proportion of participants with positive IGRA results could be covered by the current LTBI strategy in South Korea. Therefore, expansion of LTBI strategy by identifying further high-TB risk group among the general population is required.

## Data availability statement

The datasets presented in this article are not readily available because Korea Disease Control and Prevention Agency (KDCA) and National Health Insurance Service of Korea (NHIS) owns all datasets. The data used in the current study are available only after the permission from the KDCA and NHIS in advance. Requests to access the datasets should be directed to kimjusang@catholic.ac.kr.

## Ethics statement

The present study protocol was reviewed and approved by the Institutional Review Board (IRB) of Incheon St. Mary's Hospital, the Catholic University of Korea (IRB No. OC19 ZESE0023). Korea Disease Control and Prevention Agency collected informed consent from all participants when they were enrolled according to Tuberculosis Prevention Act. The study was conducted in accordance with the Declaration of Helsinki.

## Author contributions

JK, HK, HY, and J-PM designed the study. HC, HI, AP, and MJ contributed to data collection. HK and YL cleaned and verified the dataset and did the statistical analysis. JM, JC, AS, HJ, SB, H-KK, S-SL, and JP interpreted the results. HK wrote the manuscript. JK and JM reviewed and edited the manuscript. JK supervised the work. All authors had full access to all the data in the study and had final responsibility for the decision to submit for publication.
